# Differentiating SIADH from Cerebral/Renal Salt Wasting: Failure of the Volume Approach and Need for a New Approach to Hyponatremia

**DOI:** 10.3390/jcm3041373

**Published:** 2014-12-08

**Authors:** John K. Maesaka, Louis Imbriano, Joseph Mattana, Dympna Gallagher, Naveen Bade, Sairah Sharif

**Affiliations:** 1 Department of Medicine, Winthrop-University Hospital, Mineola, NY 11501, USA; E-Mails: limbriano@winthrop.org (L.I.); jmattana@winthrop.org (J.M.); nbade@winthrop.org (N.B.); ssharif@winthrop.org (S.S.); 2 Department of Medicine, Columbia University, New York, NY 10027, USA; E-Mail: dg108@columbia.edu

**Keywords:** hyponatremia, renal salt wasting, fractional excretion urate (FEurate), algorithm

## Abstract

Hyponatremia is the most common electrolyte abnormality. Its diagnostic and therapeutic approaches are in a state of flux. It is evident that hyponatremic patients are symptomatic with a potential for serious consequences at sodium levels that were once considered trivial. The recommendation to treat virtually all hyponatremics exposes the need to resolve the diagnostic and therapeutic dilemma of deciding whether to water restrict a patient with the syndrome of inappropriate antidiuretic hormone secretion (SIADH) or administer salt and water to a renal salt waster. In this review, we briefly discuss the pathophysiology of SIADH and renal salt wasting (RSW), and the difficulty in differentiating SIADH from RSW, and review the origin of the perceived rarity of RSW, as well as the value of determining fractional excretion of urate (FEurate) in differentiating both syndromes, the high prevalence of RSW which highlights the inadequacy of the volume approach to hyponatremia, the importance of changing cerebral salt wasting to RSW, and the proposal to eliminate reset osmostat as a subtype of SIADH, and finally propose a new algorithm to replace the outmoded volume approach by highlighting FEurate. This algorithm eliminates the need to assess the volume status with less reliance on determining urine sodium concentration, plasma renin, aldosterone and atrial/brain natriuretic peptide or the BUN to creatinine ratio.

## 1. Introduction

Hyponatremia, defined as serum sodium <135 mEq/L, is the most common electrolyte abnormality encountered worldwide and is an independent risk factor for higher morbidity and mortality rates [1,2]. Symptoms related to hyponatremia have been traditionally associated with severe hyponatremia and acute reductions in serum sodium, but there is a growing awareness that even mild hyponatremia is associated with mental dysfunction, unsteady gait, osteoporosis, increased falls and bone fractures [3,4,5,6,7,8,9]. Based on this awareness, there is an evolving tendency to treat every patient with hyponatremia. This recommendation creates an urgent need to assess with assurance the cause of the hyponatremia in a group of patients with diverse clinical associations and different therapeutic goals. Unfortunately, the present volume approach to hyponatremia, which has been in existence for decades, has been inadequate and misleading, in part because of misconceptions that are unsubstantiated by supportive data. Foremost among the misconceptions is the common but unproven perception that cerebral salt wasting (CSW) is a rare clinical entity. Clarification of cerebral, or the more appropriate term, renal salt wasting (RSW), *vide infra*, and its differentiation from SIADH becomes critical because of opposing therapeutic goals, which are to provide salt and water to a volume depleted patient with RSW and water restriction for a water-loaded patient with SIADH. We intend to briefly discuss the pathophysiology of RSW and SIADH, current methods of differentiating SIADH from RSW, the failure of the volume approach to address hyponatremia which has resulted in misconceptions and mismanagement of many hyponatremic patients, present data to support our proposal to change CSW to RSW, and removing reset osmostat (RO) as a subtype of SIADH, and present an algorithm which eliminates the need to assess volume, determine urine sodium concentration (UNa), plasma renin, aldosterone or atrial/brain natriuretic peptide (A/BNP).

## 2. Pathophysiology of RSW and SIADH and Evolution of Controversy on Rarity of Cerebral Salt Wasting

The initiation of RSW starts with the stimulation of a natriuretic factor that reduces sodium transport to induce RSW and extracellular volume (ECV) depletion, which in turn stimulates secretion of antidiuretic hormone (ADH), renin and aldosterone and decreases atrial/brain natriuretic peptide (A/BNP). The volume stimulus for ADH secretion is common to any state where there is ineffective circulatory volume be it heart failure or true volume depletion. The volume stimulus is more potent than the osmolar stimulus so a volume depleted patient continues to secrete ADH despite becoming progressively hyponatremic as long as the patient continues to take in free water [10]. Removal of the volume stimulus allows the coexistent hypo-osmolality to inhibit ADH secretion, remove water from the body by excreting dilute urines and correcting the hyponatremia to illustrate appropriate ADH secretion in RSW [11,12]. As previously reviewed, the first descriptions of CSW failed to prove with certainty a salt wasting syndrome [13,14]. RSW was possible in one patient who was described as being dehydrated with a urine chloride of 61.6 mEq/L [13]. Previous studies have demonstrated that a sodium depleted patient will virtually eliminate sodium from urine until the sodium losses have been replaced [15,16,17]. The high urine chloride and presumably sodium concentration in urine would, thus, have been consistent with RSW and the term CSW was thus derived.

SIADH evolved as a clinical entity by the demonstration of a clinical correlate to the seminal work by Leaf *et al.*, who defined the consequences of administering vasopressin to normal human subjects [18]. The proposal of an inappropriate secretion of ADH in the absence of methods to determine plasma ADH levels epitomized the application of basic physiologic principles to the bedside [19]. This hypothesis was later proven by demonstrating inappropriately high ADH levels that did not respond to the usual volume and osmolar stimuli [19]. Moreover, ECV measured by the sulfate method in the first reported case of SIADH proved that it was possible for a euvolemic/hypervolemic patient to have hyponatremia with high UNa without implicating a renal salt wasting syndrome [19]. An increase in intravascular volume by the gold standard radioisotope dilution method has been demonstrated by others in SIADH [12,20,21]. It became evident that the original case of CSW in a “dehydrated” patient with high UNa could have had SIADH because of the general agreement that one cannot accurately assess volume status by usual clinical criteria. The existence of CSW, therefore, was seriously questioned to the point of being considered either nonexistent or certainly rare.

## 3. Differentiating SIADH from RSW

Differentiating SIADH from RSW has been extremely difficult to accomplish, in part because of significant overlapping clinical findings between both syndromes. As noted in Table 1, both syndromes are associated with intracranial diseases, have normal renal, thyroid and adrenal function, are hyponatremic and hypouricemic and have concentrated urines, high UNa over 40 mEq/L, and high fractional excretion (FE) of urate. The only clinical difference is the state of their ECV, being euvolemic or hypervolemic in SIADH and hypovolemic in RSW. The overlapping of major clinical characteristics between SIADH and RSW as noted in Table 1 and the perception that RSW is a rare clinical entity have virtually eliminated RSW from consideration at the bedside. This diagnostic dilemma needs to be urgently resolved because of the evolving awareness that even mildly hyponatremic patients are symptomatic and should therefore be treated [5,7]. These perceptions and recommendations are in large part influenced by reports of unsteady gait and a fourfold increase in fall rates which appears to be consistent from a serum sodium of 115–132 mEq/L. In addition, a fourfold increase in bone fractures in elderly hyponatremic patients and increased osteoporosis with chronic hyponatremia has been reported [5,7,22]. The urgency in resolving the diagnostic and therapeutic dilemma becomes most evident by the divergence in therapeutic goals of water restricting patients with SIADH and administering salt and water in RSW.

**Table 1:** List of features common to syndrome of inappropriate antidiuretic hormone secretion (SIADH) and renal salt wasting (RSW), except divergent volume status.

**Clinical Findings Common to both SIADH and RSW**
Association with intracranial disease
Hyponatremia
Concentrated urine
Urine sodium [Na] usually >20 mEq/L
Non-edematous
Hypouricemia, with increased fractional excretion urate (FEurate)
**Only Difference between SIADH and RSW**
Volume state: normal/high in SIADH
low in RSW

There is universal agreement that we cannot assess ECV with any degree of accuracy by usual clinical criteria, yet the approach to hyponatremia starts with an assessment of volume. The ineffectiveness of this volume approach is becoming even more evident by an objective review of the literature and recent publications of RSW occurring in patients without clinical cerebral disease [11,12]. Since an assessment of volume is essential in differentiating SIADH from RSW, let us review studies that have determined volume by credible methods. As noted in Table 2, 83%–94% of hyponatremic patients with different forms of neurosurgical diseases were found to have hypovolemia with high UNa that met the criteria for RSW as compared to hypervolemic patients with SIADH. Blood volume was determined by gold standard radioisotope dilution methods, including 51 chromium labeled red cells and/or radio iodinated serum albumin (RISA) [20,21,23]. It is clear from these studies that RSW is much more common than SIADH, yet it is still perceived as a rare clinical entity, which has been propagated for many years without either negating these compelling studies nor by providing evidence to the contrary by suitable methods. Moreover, water restricting these patients for an erroneous diagnosis of SIADH when in fact they have RSW has been reported to increase morbidity and mortality rates in patients with subarachnoid hemorrhage [12,24,25]. These data emphasize the importance of differentiating SIADH from RSW in order to develop better targeted therapeutic strategies.

**Table 2:** Summary of volume studies by gold standard radio-isotope dilution methods in hyponatremic neurosurgical patients. (Note: RSW is much more common than SIADH).

Author [ref.]	No. of Patients	Low Blood Volume RSW	Increased Blood Volume SIADH	Urine Na mEq/L
Nelson [20]	12	10 (83%)	2	41–203
Wijdicks [21]	9	8 (89%)	1	--
Sivakumar [23]	18	17 (94%)		43–210

## 4. Value of Determining FEurate in Differentiating SIADH from RSW and Changing CSW to RSW

**Figure 1:**
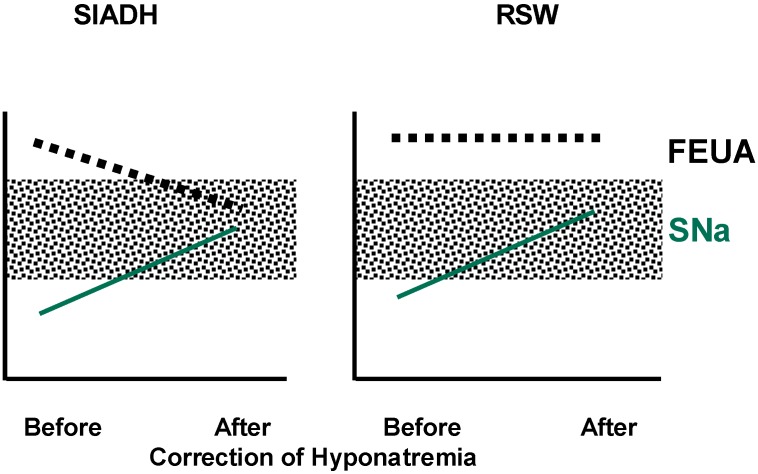
Changes in FEurate in SIADH and RSW after correction of hyponatremia. Shaded areas represent normal ranges. (Maesaka J. K., modified from [14]).

Calculation of fractional excretion (FE) of urate determines the percent excretion of the filtered load of urate at the glomerulus. It determines the net transport of urate without distinguishing what is secreted or reabsorbed and can be readily determined by collecting blood and spot urine at the same time. FEurate in percentage excretion of the filtered load of urate can be determined by dividing the ratio of urine to plasma urate by the ratio of urine to plasma creatinine and multiplying by 100. It can also be derived by dividing the product of UNa (×) serum urate by the product of serum sodium (×) urine creatinine and multiplying by 100. FEurate, normal 4%–11%, has been consistently increased to >11% in SIADH and RSW and has a unique relationship to serum sodium in SIADH and RSW. In SIADH, correction of hyponatremia will normalize FEurate to 4%–11% as compared to being persistently increased to >11% in RSW, Figure 1 [26,27,28,29]. The relationship between FEurate and serum sodium can now be utilized to differentiate SIADH from RSW by correcting the hyponatremia by any means, be it by water restriction or isotonic/hypertonic saline and observing whether FEurate normalizes to 4%–11% as in SIADH or remains increased above 11% in RSW, Figure 1 [11,12,30,31,32,33]. FEurate can exceed normal values in patients with reduced GFR, so the algorithm is valid in patients with serum creatinine less than 1.5 mg/dL.

**Figure 2:**
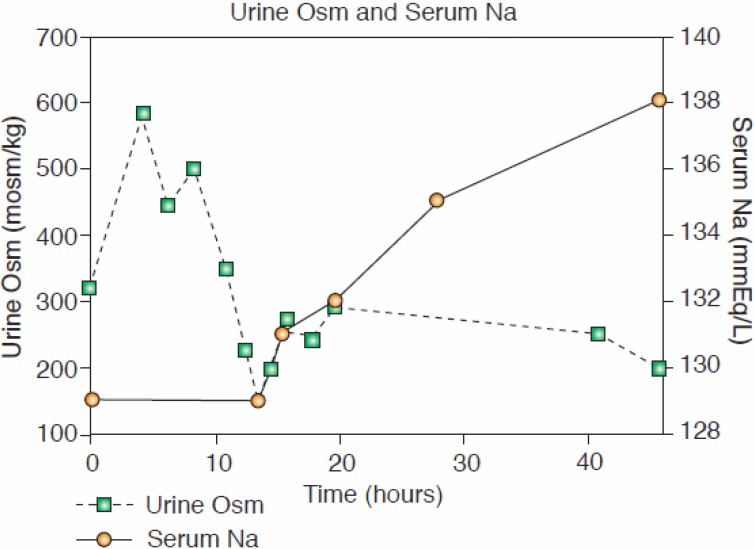
Correction of serum sodium and achievement of dilute urine after saline infusion (with permission, *Kidney International*, Maesaka J. K. [12]).

Two unequivocal cases of RSW without clinical evidence of cerebral disease not only verified the persistent increase in FEurate with correction of hyponatremia, but served as the basis for us to make a clinically important proposal to change CSW to RSW [11,12,34]. One was a hyponatremic patient with a simple hip fracture with no clinical evidence of cerebral disease who exhibited all of the essential features of RSW. The hyponatremia was associated with a 7% reduction in blood volume as determined by the gold standard radioisotope dilution method, using 51 chromium labeled red blood cells and RISA, increased baseline plasma renin, aldosterone, and ADH and low normal atrial natriuretic peptide. She diluted her urine to 170 mOsm/kg 13 h after initiation of saline therapy when her plasma ADH level was not detectable. Her serum sodium increased to 138 mEq/L within 48 h (Figure 2), and the increased FEurate persisted with normalization of serum sodium (Figure 1) [12]. A similar case of RSW was a hyponatremic patient with a pneumonia without cerebral disease, who diluted his urine 20 h after initiating saline therapy [11]. As in the hip fracture patient, the increased FEurate persisted after volume repletion and correction of hyponatremia. A water loading test was normal after volume repletion and correction of his hyponatremia, suggesting that like the patient with a hip fracture, there was an appropriate hypovolemia-induced increase in ADH. Saline infusions eliminated the volume stimulus for ADH secretion to allow the coexistent plasma hypo-osmolality to inhibit ADH secretion, thereby excreting dilute urines and correcting the hyponatremia [11,12]. In two patients with SIADH and increased blood volumes, saline infusion failed to dilute their urine or correct their hyponatremia [11].

The relationship between serum sodium and FEurate as noted in Figure 1 may be the best available method to differentiate SIADH from RSW. Correction of the hyponatremia can be accomplished by any method, including hypertonic saline, to differentiate SIADH from RSW. Many consider the administration of saline to be a detriment to the evaluation of the hyponatremic patient because of its effect on UNa and plasma renin, aldosterone and A/BNP. As extensively reviewed in our review of renal urate transport, we cite four papers that demonstrate the meager effect of saline infusions on FEurate, Table 3 [35,36,37,38]. The meager effect of saline on FEurate can be illustrated in a hyponatremic patient with high FEurate who posed a diagnostic dilemma of not being able to determine whether the patient had SIADH or RSW. The diagnosis of SIADH was made by increasing serum sodium to 138 mEq/L by administering liberal amounts of isotonic and hypertonic saline and observing a marked decrease in FEurate from 26% to 8% [32]. The marked reduction in FEurate after the administration of isotonic and hypertonic saline is contradictory to the common belief that saline reduces the net transport of many solutes, including urate. The reduction in FEurate after correction of hyponatremia by isotonic and hypertonic saline raises an intriguing question as to why FEurate would normalize in SIADH and remain increased in RSW. As previously reviewed, the increase in FEurate cannot be explained by the V1 activity of ADH or chronic hyponatremia in SIADH but it is probable that the natriuretic factor demonstrated in RSW might reduce urate transport in the proximal tubule where urate is exclusively transported and is the major site of inhibiting sodium transport by the natriuretic factor [39,40,41].

**Table 3:** Summary of extracellular volume expansion with isotonic, hypotonic and hypertonic saline on fractional excretion of sodium [FEsodium] and urate [FEurate] at control and experimental (Exp.) periods after saline administration.

	FE Na (%)		FE Urate (%)		Reference
	Control	Exp.	Control	Exp.	
Isotonic	1.04	4.43	7.98	9.76	[37]
	1.6	8.2	5.0	5.8	[36]
Hypertonic	2.9	18.6	5.4	12.1	[36]
	1.4	14.5	12.5	18.7	[35]
Hypotonic	1.1	6.1	4.0	7.3	[36]

## 5. Normal FEurate Identifies Patients with Reset Osmostat

The value of determining FEurate in hyponatremic conditions has been further amplified by a normal FEurate being observed in every hyponatremic patient with RO [42]. The normal FEurate seen in psychogenic polydipsia and possibly in beer potomania can be readily identified by the history of excess intake of water or beer, respectively [42,43]. Based on these data, we proposed eliminating RO as type C SIADH because of the normal FEurate, which is pathophysiologically different from the high FEurate seen in SIADH, and a predictable response to water-loading [42].

## 6. Proposal of New Algorithm

**Figure 3:**
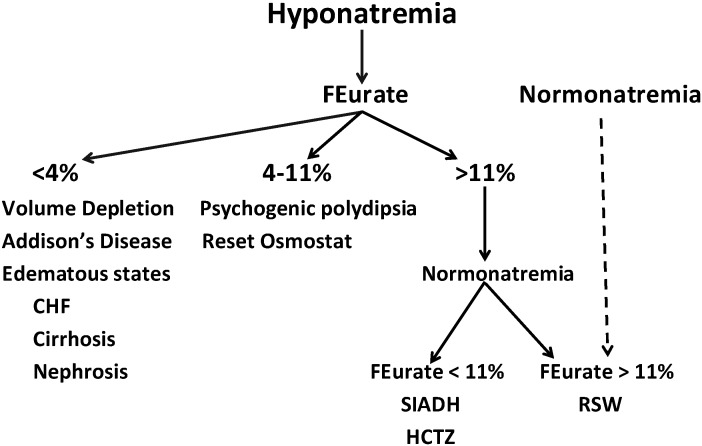
Algorithm for determining cause of hyponatremia, using FEurate.

Based on a large database, we would like to introduce a new, updated algorithm which centers on the determination of FEurate outlined in Figure 3. Each citation can be supported by credible data. This algorithm has been found to be superior to the traditional volume approach, which has been used for decades and is clearly inadequate. Once the patient has been identified to have hyponatremia, defined usually to be a serum sodium <135 mEq/L, we suggest determining FEurate by obtaining a spot blood and urine sample and utilizing the formula provided above. The administration of saline should not affect the FEurate to any significant degree (Table 3), and drugs such as atorvastatin and losartan which are known to increase urate excretion have not been shown to have a meaningful effect on the results [44,45]. If the FEurate exceeds 11%, it is consistent with either SIADH or RSW but repeating FEurate after correction of hyponatremia by any method, such as water restriction or administration of hypertonic saline, will differentiate SIADH from RSW; it will normalize to 4%–11% in SIADH and remain >11% in RSW. If the FEurate is normal, between 4% and 11%, it is most consistent with RO but can be seen in psychogenic polydipsia [43,44,46,47], and possibly in beer potomania. Psychogenic polydipsia can be readily diagnosed by the history of ingesting large volumes of water, having polyuria and excretion of dilute urines, and beer potomania by the history of ingesting large amounts of beer with low solute intake [42,43]. If the FEurate is <4%, it is consistent with pre-renal conditions including volume depleted states or inadequate perfusion with normal renal function as in true volume depletion, edematous states such as congestive heart failure, cirrhosis, nephrosis and pre-eclampsia [48]. The low FEurate in Addison’s disease can be explained by salt wasting due to a distal defect in sodium transport where mineralocorticoids exert their effect. The intact proximal tubule would thus increase the reabsorption of urate and other solutes as is typical of pre-renal azotemia, FEurate <4% [48]. The dotted lines connecting a high FEurate with normonatremia and RSW can be supported by indirect data but it is our belief that this will eventually turn out to be a predictor of RSW without going through a phase of hyponatremia because the patient had very little water intake. This possibility is evident by the need to ingest water to become hyponatremic, since the insensible water losses are largely hypotonic to induce hypernatremia without sufficient water intake. Developing hyponatremia without water intake is extremely difficult if not impossible to achieve except if UNa exceeds serum sodium concentration in the absence of water intake.

This algorithm omits a reliance on plasma renin, aldosterone, A/BNP, UNa or even the BUN to creatinine ratio. Plasma renin, aldosterone and A/BNP have not been consistently reliable indices because of the effect of saline infusions that are often utilized in hyponatremic patients, the presence of chronic kidney disease and frequent use of ACE inhibitors, angiotensin receptor blockers, diuretics and beta blockers. The determination of UNa has been utilized to evaluate patients with hyponatremia, but UNa <20 and even <40 mEq/L are being observed with greater frequency so this parameter has not proven to be effective in the evaluation of hyponatremic conditions. The low UNa in our experience has been observed much more commonly in patients with RSW as compared to patients with SIADH and RO. Since UNa reflects salt intake in conditions such as SIADH, RO and RSW, the low UNa suggests that these subjects have reduced intake of salt, probably reflecting the severity of their co-morbid condition. In some cases, the reduced salt intake could be iatrogenic. This has been reported with increased morbidity when the patient with RSW was fluid restricted for an erroneous diagnosis of SIADH [12,24,25,30]. This was the case in our RSW patient with a hip fracture whose UNa was 6 mEq/L after being fluid restricted for 10 days for an erroneous diagnosis of SIADH [12,24,25]. The determination of UNa has not been useful in the evaluation of patients with hyponatremia.

There is mounting evidence to prove the ineffectiveness of the volume approach to hyponatremia. Surprisingly, about a third of hyponatremic patients outside of the neurosurgical intensive care unit had RSW; the majority demonstrating no clinical evidence of cerebral disease; thus providing additional support for our proposal to change CSW to RSW [34,49]. As discussed, this is an important change, as RSW would otherwise not be considered in the absence of clinical cerebral disease [34]. The high prevalence of RSW was noted in a population where the “clinical” volume approach would have been attributed to SIADH because of the perception that SIADH is the most common cause of hyponatremia in this and all other clinical settings. This raises the possibility that the high morbidity and mortality rates associated with hyponatremia may have a significant iatrogenic component secondary to inappropriate water restriction in patients with RSW who were thought to have SIADH. These data provide further evidence for the ineffectiveness of the volume approach to evaluating patients with hyponatremia and support the common notion that we cannot assess the volume status of patients with any degree of accuracy. To persist in this outmoded approach will lead to misdiagnosis and mistreatment of patients with hyponatremia that will lead to increased morbidity and mortality of a group of patients with what appears to be more serious co-morbid conditions. The proposed algorithm has been found to be superior to the “clinical” volume approach and should be tested by other groups for its clinical effectiveness until newer algorithms can be developed.

## 7. Conclusions

It is extremely difficult to differentiate SIADH from RSW largely because of significant overlapping of clinical and laboratory findings and the perception that RSW is a rare clinical entity. This differentiation is extremely important because of divergent therapeutic goals of appropriately water restricting those with SIADH and increasing salt and water with RSW to avoid iatrogenic increases in morbidity and mortality. The recent recommendations to treat most or all patients with hyponatremia introduce an urgency to resolve this diagnostic and therapeutic dilemma. Changing CSW to RSW is an important modification in nomenclature that will expand our consideration of a large number of RSW patients without evidence of clinical cerebral disease and to avoid mismanagement and possibly reduce morbidity and mortality. The volume approach to hyponatremia and perception that RSW is a rare clinical entity should be abandoned in favor of a more open-minded approach that will lead to better diagnosis and treatment of hyponatremic conditions. To this end, we propose a new algorithm that utilizes FEurate as a pivotal determination, (Figure 3). Determining FEurate after correcting hyponatremia by judicious use of hypertonic saline might be an effective way of differentiating SIADH from RSW (Figure 1), being mindful of avoiding too rapid correction of hyponatremia to reduce the risk of developing osmotic demyelination, monitor the patient for any evidence of fluid overload such as induction of heart failure and that saline has a meager effect on FEurate. The new algorithm eliminates the determination of plasma renin, aldosterone and A/BNP and UNa, which have been found to be ineffective and often misleading.
